# Effect of Blended Perfluorinated Sulfonic Acid Ionomer Binder on the Performance of Catalyst Layers in Polymer Electrolyte Membrane Fuel Cells

**DOI:** 10.3390/membranes13090794

**Published:** 2023-09-13

**Authors:** Beom-Seok Kim, Jong-Hyeok Park, Jin-Soo Park

**Affiliations:** 1 Department of Green Chemical Engineering, College of Engineering, Sangmyung University, Cheonan 31066, Republic of Korea; kbs8762@gmail.com; 2 Department of Civil, Environmental and Biomedical Engineering, The Graduate School, Sangmyung University, Cheonan 31066, Republic of Korea; sbq6358@gmail.com; 3 Future Environment and Energy Research Institute, Sangmyung University, Cheonan 31066, Republic of Korea

**Keywords:** perfluorinated sulfonic acid ionomer, blended ionomer, proton conductivity, catalyst layer, polymer electrolyte fuel cell

## Abstract

In this study, blended perfluorinated sulfonic acid (PFSA) ionomers with equivalent weights (EWs, g/mol) of ~1000, 980, and 830 are prepared. Catalyst layers (CLs), using blended PFSA ionomers, with different side chain lengths and EWs are investigated and compared to CLs using single ionomers. The ion exchange capacity results confirm that blended ionomers have the target EWs. As a result, blended ionomers exhibit higher ion conductivity than single ionomers at all temperatures due to the higher water uptake of the blended ionomers. This implies that blended ionomers have a bulk structure to form a competent free volume compared to single ionomers. Blended ionomers with short side chains and low EWs can help reduce the activation energy in proton conduction due to enhanced hydrophobic and hydrophilic segregation. In addition, when using the blended ionomer, the CLs form a more porous microstructure to help reduce the resistance of oxygen transport and contributes to lower mass transfer loss. This effect is proven in fuel cell operations at not a lower temperature (70 °C) and full humidification (100%) but at an elevated temperature (80 °C) and lower relative humidity (50 and 75%). Blended ionomer-based CLs with a higher water uptake and porous CL structure result in improved fuel cell performance with better mass transport than single ionomer-based CLs.

## 1. Introduction

Global fossil fuel consumption has rapidly increased due to massive industrialization, which has a huge impact on the environment, such as climate change caused by greenhouse gas emissions [1]. The utilization of clean energy from new and renewable sources is considered to be an effective solution for the resolution of both the shortage of electrical energy and the reduction in greenhouse gas emissions. Hydrogen is an emerging energy source used to solve energy and environmental problems because electrical energy can be produced from the electrochemical reactions between hydrogen and oxygen. Recently, various production technologies of hydrogen are being developed for massive supply to fuel cells [2,3].

Polymer electrolyte membrane fuel cell (PEMFC) is commonly used as an energy conversion device from hydrogen to electrical energy since it has the advantage that its conversion efficiency is significantly higher than that of conventional power generation devices [4,5,6]. PEMFC technology is taking a step towards being a competitor of incumbent and emerging technologies across multiple applications. This competitiveness is supported by a cost reduction and no performance and durability loss. In other words, the performance and durability of PEMFC technology under commercially viable operating conditions, such as a high temperature (>70 °C) and ~50% relative humidity (R.H.), should be enhanced at the same time even though they are in a trade-off relationship. To achieve this goal, system-level competitiveness is a priority, and component and stack level R&D endeavors are simultaneously in development. Catalyst layers (CLs) could be optimized by using sophisticated and integrated research efforts such as the control of the CL morphology via ink composition, concentration, solvent, functionalization of electrocatalyst, and fabrication to attain higher Pt utilization and oxygen transport. In order to maximize the performance of PEMFC, three main irreversible losses, i.e., activation, Ohmic, and mass transport overpotential, should be minimized as the CL of PEMFC, where the main electrochemical reactions, such as the oxidation of hydrogen and the reduction of oxygen, take place at various operating conditions. Both CLs are attached onto a piece of polymeric electrolyte membrane to complete membrane–electrode assembly (MEA) to allow protons to be transported through the membrane and electron via an external circuit for complete fuel cell reactions. Most of the activation overpotential in PEMFC originates from the result of an oxygen reduction reaction (ORR) at the cathode:

O_2_ + 4H^+^ + 4e^−^ → 2H_2_O

In a previous report, it was also found that the ORR in CLs is primarily limited by mass transport resistance [7,8]. Therefore, it is necessary to optimize the microstructure of CLs at the cathode for an improved ORR, which forms a sufficiently thick ionomer shell, exhibiting a highly aggregated phase morphology, and retains sufficient void space for unimpeded gas transport [9]. Ionomers are commonly utilized as proton conductors in CLs for MEAs to facilitate the activation of electrochemical reactions at the triple phase boundary (TPB) through proton conduction as well as hydrogen and oxygen permeation. The performance of CLs is highly influenced by platinum on a carbon-supported catalyst and ionomer formation structure. Thus, the pore size and porosity of CLs differ with respect to the type of catalyst and ionomer [10,11,12,13].

Perfluorosulfonic acid (PFSA) ionomers, such as Nafion^®^ or Aquivion^®^, are used as the membrane and ionomer binder in MEAs due to good proton conductivity at low water uptake, chemical stability, and mechanical stability [14]. PFSA typically consists of hydrophobic Teflon-like backbones and hydrophilic sulfonate (SO_3_^−^)-bearing side chains, which conduct protons through a crystalline phase-segregated structure with localized hydrophobic and hydrophilic domains [15,16,17,18]. The PFSA membrane acts as a bulk proton-conducting membrane, while the ionomer in CLs is achieved by a thin film being coated on a catalyst and/or catalyst support particles, as well as agglomerates, and filling the regions between aggregates [9]. Nafion^®^ is an ionomer with long side chains (LSCs) consisting of five −CF_2_ groups. Aquivion^®^ is a short side chain (SSC) ionomer with two −CF_2_ groups. These ionomers are most widely used as ion-conductive polymers in the CLs of MEAs [19,20].

PFSA ionomers play an important role in CLs to determine the microstructure, consisting of pores, an ionomer coated shell on catalyst particles, and ionomer aggregates. The structure of CLs could be determined by the length of PFSA side chains and the equivalent weight (EW, g/mol). The performance of the MEA is therefore influenced by the microstructure of CLs [21,22,23]. For instance, it was reported that utilizing SSC ionomers could help reduce the formation of dense ionomer layers that impede O_2_ transport and catalyst activity compared to LSC ionomers [24,25]. Moreover, LSC-PFSA and SSC-PFSA ionomers were utilized to investigate the mass transport behavior in the MEA during PEMFC operation. SSC-PFSA ionomer-based CLs demonstrated higher proton conductivity and lower gas transport resistance compared to LSC-PFSA ionomer-based CLs due to a beneficial pore structure that enhanced the electrochemical reaction. This looser porous structure with larger porosity, in contrast to LSC-PFSA ionomer-based CLs, contributed to the high performance of MEAs [26,27,28]. Nafion^®^ and Aquivion^®^ were also utilized as binders for CLs as well as solid electrolytes for MEA in PEMFCs. Currently, Nafion^®^ ionomers such as D2021 (EW 1100) and D2020 (EW 1000) are being used as membranes and binders, respectively. Aquivion^®^ ionomers, named D72-25BS (EW 720), D98-25BS (EW 980), and D83-24B (EW 830), are widely used. The results were evaluated in terms of the short- and long-term performance of CLs using SSC- or LSC-PFSA ionomers. The SSC-PFSA ionomer showed a better performance because of a higher ion exchange capacity (IEC) and water uptake capacity than the LSC-PFSA ionomer. However, the SSC-PFSA ionomer exhibited higher degradation than the LSC-PFSA ionomer, indicating the influence of the length of the CF_2_ chain. In other words, the LSC is chemically stable and can function as both a binder and a proton conductor in CLs for long-term applications [13,23,29,30]. Despite the advantages for CLs using a single type of ionomer, studies of CLs utilizing blended ionomers are lacking [31,32]. To understand the effect of blended ionomers on CLs, ionomer aggregates, ionomer–catalyst particle interaction, and the thin film structure of ionomers on catalysts and support particles should be studied.

In this work, CLs using blended PFSA ionomers with different side chain lengths and EWs are investigated to understand how blended ionomers form thin films in CLs and how the thin films influence the CL performance compared to CLs utilizing single PFSA ionomers. Blended ionomer dispersions were prepared by mixing Nafion EW 1100 and Aquivion EW 720 as well as Aquivion EW 980 and Aquivion EW 720 to prepare LSC/SSC blended-EW 1000 and -EW 980 and SSC/SSC-EW 830. To investigate the blending effect on CL performance, CLs using various types of blended ionomers were prepared and evaluated. The physicochemical properties and electrochemical performance of single and blended ionomers and CLs were characterized in terms of proton conductivity, water uptake, IEC, pore size distribution, voltammetry, and current-voltage (I-V) polarization.

## 2. Materials and Methods

### 2.1. Materials

The Nafion D1021 (10 wt.% in water, EW 1100, Chemours, DE, USA) and Nafion D2020 (20 wt.% in water/alcohol, EW 1000, Chemours, DE, USA) as an LSC ionomer and Aquivion D98-25BS (25 wt.% in water, EW 980, Solvay, Brussels, Belgium), Aquivion D83-24BS (24 wt.% in water, EW 830, Solvay, Brussels, Belgium), and Aquivion D72-25BS (25 wt.% in water, EW 720, Solvay, Brussels, Belgium) as an SSC ionomer were used as received for the solution-cast membrane and CL binder for the measurement of membrane properties and CL performance in PEMFCs. *N*,*N*-dimethylacetamide (DMAc) (99%, JUNSEI Chemical Co., Tokyo, Japan) was used as a solvent to disperse solid ionomer and to prepare a solution-cast membrane. TKK TEC10F50E (47.0 wt.% Pt, TANAKA, Tokyo, Japan) as an electrocatalyst and deionized water, 1-propanol (CARLO ERBA Reagents, Barcelona, Spain), and 2-propanol (CARLO ERBA Reagents, Barcelona, Spain) as solvents were used. Nafion 212 (50 µm, Chemours, Wilmington, DE, USA) was used as a common electrolyte membrane in MEA.

### 2.2. Preparation of Blended Ionomer Dispersion

Three different types of blended ionomer dispersions were prepared with EWs of ~1000, ~980, and ~830 made of Nafion D1021 (EW 1100) and Aquivion D72-25BS (EW 720) with a weight ratio of 7.5:2.5, Nafion D1021 (EW 1100) and Aquivion D72-25BS (EW 720) with a weight ratio of 7.0:3.0, and Aquivion D98-25BS (EW 980) and Aquivion D72-25BS (EW 720) with a weight ratio of 4.5:5.5, respectively. Nafion D2020 (EW 1000), Aquivion D98-25BS (EW 980), and Aquivion D83-24B (EW 830), which were commercially available ionomer dispersions, were used to make a comparison to the aforementioned blended ionomers. The ratios of the blended ionomers for the theoretical EWs, i.e., EW 1000, EW 980, and EW 830, were calculated as follows:

(EW 1100 × 0.75) + (EW 720 × 0.25) ≒ EW 1000 (EW 1100 × 0.70) + (EW 720 × 0.30) ≒ EW 980 (EW 980 × 0.45) + (EW 720 × 0.55) ≒ EW 830

### 2.3. Preparation of Solution-Cast Membrane

Blended and single ionomers were obtained from the blended and commercially available ionomer dispersions by evaporating solvents using a convective oven at 60 °C for 24 hrs. All the ionomers were solubilized at 0.2 g/mL in anhydrous DMAc. The ionomer solutions were dispersed for 1 h and then cast on a glass plate using a doctor blade. The coated plate was heated at 120 °C for 24 h and annealed at 190 °C for 3 h in a convective oven. The prepared membranes with thicknesses of ~50 µm were equilibrated in 1 mol/L HCl (37.0%, JUNSEI Chemical Co., Tokyo, Japan) solution for 24 h at room temperature (R.T.). Then, the membranes were rinsed in deionized water several times prior to measurement [33].

### 2.4. Preparation of CLs and MEAs

Commercially available SSC and LSC ionomers and the three types of the blended ionomer dispersions were used as the binder for the preparation of CLs for MEAs. The components of all catalyst inks are each ionomer, electrocatalyst (TKK TEC10E50E), and extra solvents (1-propanol, 2-propanol, and deionized water). The solid content of all catalyst inks was 4.0 wt.%, and the ratio of ionomer/carbon (I/C) was 0.9. The catalyst ink was homogenized via sonication and magnetic stirring for 30 min and 24 h, respectively, followed by direct coating on both sides of a Nafion 212 membrane by spraying with 0.4 mg_pt_/cm^2^ and 9 cm^2^ active area [33,34]. The averaged thickness of the prepared CLs was approximately 20 μm, measured by a digital thickness gauge (H-2781, Mitutoyo, Kawasaki, Japan).

### 2.5. Characterization

#### 2.5.1. IEC

IEC was measured by titration. Prepared membranes using all the ionomer dispersions were immersed in 1.0 M HCl to convert exchangeable groups into protons for 24 h. The pretreated membranes were soaked in 1.0 M NaCl (99.5%, JUNSEI Chemical Co., Tokyo, Japan) for 24 h to exchange protons into sodium. The proton in the NaCl solution was titrated by using a titration instrument (848 Titrino plus, Metrohm, Herisau, Switzerland) with a 0.01 M NaOH (99.9%, JUNSEI Chemical Co., Tokyo, Japan) solution [33]. The IEC was calculated as:

$$\mathrm{IEC}\left(\frac{\mathrm{mol}}{g}\right)=\frac{C_{\mathrm{NaOH}}\times \left(V_{\mathrm{eq}}-V_{\mathrm{blank}}\right)}{W_{0}}$$

where W_0_ is the dry weight of the membranes, C_NaOH_ is the concentration of a NaOH solution, V_eq_ is the titrated volume of a NaOH solution for samples, and V_blank_ is the titrated volume of a NaOH solution for blanks.

#### 2.5.2. Proton Conductivity

The proton conductivity of the prepared membranes was measured using the in-plane method by a membrane conductivity cell (MCC, WonATech, Seoul, Republic of Korea) with a four-electrode system. The measurements were conducted at several temperatures with R.H. 100%, or at 80 °C with R.H. 50, 75, and 100%. The proton conductivity of the membranes was determined by impedance, which was measured in the frequency range of 100 kHz to 1 Hz with 10 mV amplitude using a potentiostat (SP-150, Bio-Logic Science Instruments, Seyssinet-Pariset, France). The Ohmic resistance (R) was determined by impedance with a zero-phase angle [34,35].

The proton conductivity was calculated as:

$$\sigma \;\left(S/\mathrm{cm}\right)=\frac{l}{R\cdot \;S}$$

where l is the distance between two working electrodes, R is the Ohmic resistance of the membranes, and S is the cross-sectional area of the membranes.

The activation energy for proton conduction was calculated via the Arrhenius equation using membrane proton conductivity with temperature.

The activation energy was calculated as:

$$\sigma \;\left(S/\mathrm{cm}\right)={\mathrm{Ae}}^{-\frac{E_{a}}{\mathrm{RT}}}$$

where σ is the proton conductivity of the membranes, A is the pre-exponential factor, T is the measured temperature (K), R is the ideal gas constant (8.314 J/mol∙K), and E_a_ is the activation energy (J/mol).

#### 2.5.3. Water Uptake

The prepared membranes were dried at 80 °C to remove water in the membranes and were then immersed in the deionized water at temperatures of R.T., 30, 50, 70, and 90 °C for 1 h. The weight of the samples was measured before and after immersing. The water uptake measurements were performed 3 times for each membrane.

The water uptake was calculated as:

$$\mathrm{Water}\;\mathrm{uptake}\;\left(W\right)\%=\frac{W_{1}-W_{0}}{W_{0}}\times 100\%$$

where W_0_ is the dry weight of the membranes and W_1_ is the wet weight of the membranes.

#### 2.5.4. Mercury Intrusion Porosimetry

The pore size and pore distribution of the prepared CLs containing blended or single ionomers were measured using a pore characterization system (AutoPore 9520, Micromeritics, Norcross, GA, USA). The prepared CL-coated membranes were diced into several pieces of 1 cm^2^ until the total amount of all diced pieces was 0.5 g. The applied pressure range was 0 to 60,000 psia [36].

#### 2.5.5. Electrochemical Characterization

Two pieces of the gas diffusion layers (JNTG 20-A3, JNT Group, Hwaseong, Gyeonggi-do, Republic of Korea) were placed on the active area of a CL-coated membrane, and the Teflon gaskets were positioned in a unit cell (CNL Co. Ltd., Seoul, Republic of Korea) with an active area of 9 cm^2^. The unit cell was mounted in the fuel cell evaluation station (CNL Co. Ltd., Seoul, Republic of Korea). I-V polarization, cyclic voltammetry (CV), and limiting current density were conducted by an electrical loader or a potentiostat.

The unit cell was operated under various conditions, i.e., 70 °C with R.H. 100%, 75 °C with R.H. 100%, and 80 °C with R.H. 50, 75, and 100% at ambient pressure. Prior to the I-V characterization, the unit cell was activated using the following steps: a voltage scan from 0.9 to 0.4 V with a scan rate of 0.05 V/s, and a constant voltage of 0.4 V for 3 min and a voltage scan from 0.4 to 0.9 V with a scan rate of 0.05 V/s. Fuel cell performance was measured in a voltage range of 0.9 to 0.3 V. Humidified hydrogen at a flow rate of 0.254 L/min and air at a flow rate of 0.805 L/min were supplied to the anode and cathode, respectively. Electrochemical impedance spectroscopy of MEAs was measured under the same condition as the I-V characterization. For the CV measurement, hydrogen and nitrogen were supplied to the anode and cathode at the same flow rate of 0.200 L/min. The voltage scan range for CV was 0.1 to 1.1 V (vs. SHE). The electrochemically active surface area (ECSA) of CLs was calculated from the CV results [35,37,38]. Limiting current density was determined from I-V polarizations measured at the anodic conditions (hydrogen, R.H. 100%, 70, 75, and 80 °C, 0.254 L/min) and the cathodic conditions (oxygen, R.H. 100%, 70, 75, and 80 °C, 0.120, 0.202, 0.402, 0.603, and 0.805 L/min) with different oxygen ratios of 14.3, 23.8, 47.6, 71.5, and 100% which correspond to the oxygen concentration in the air, i.e., 3, 5, 10, 15, and 21%.

## 3. Results and Discussion

To investigate the blending effect of SSC and LSC ionomers on CL performance, three different types of blended ionomer dispersions, i.e., LSC/SSC blended-EW 1000 and -EW 980 and SSC/SSC-EW 830, were prepared by mixing Nafion D1021 (EW 1100) and Aquivion D72-25BS (EW 720), as well as Aquivion D98-25BS (EW 980) and Aquivion EW 720, and compared with single ionomer dispersions, i.e., Nafion D2020 (EW 1000), Aquivion D98-25BS (EW 980), and Aquivion D83-24B (EW 830). The experimental EWs calculated as the reciprocal of IEC of the blended ionomers and single ionomers were compared to confirm whether the blended ionomers had similar EWs to the single ionomers prior to the evaluation of CL performance. Table 1 summarizes the experimental EW results of the blended and single ionomers. All values have a standard deviation of less than 5%. The EW results indicate that the blended ionomers have similar targeted EWs so as to compare them with the single ionomers.

**Figure 1:**
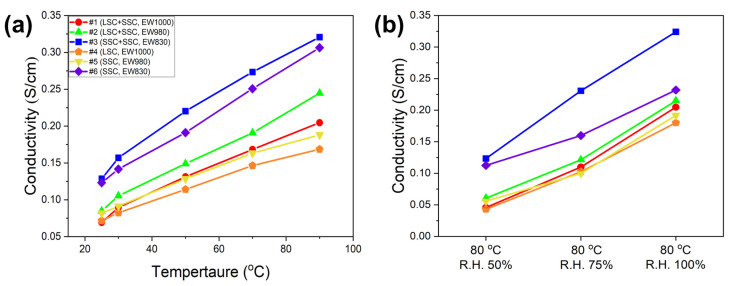
The ion conductivity of the blended #1 (circle, LSC + SSC, EW 1000), #2 (triangle, LSC + SSC, EW 980), and #3 (square, SSC + SSC, EW 830) ionomers and the single #4 (pentagon, LSC, EW 1000), #5 (inverted triangle, SSC, EW 980), and #6 (diamond, SSC, EW 830) ionomers as a function of (**a**) temperature at R.H. 100% and (**b**) R.H at 80 °C.

The ion conductivity of the ionomers was evaluated at R.T., 30, 50, 70, and 90 °C with R.H. 100% and at 80 °C with R.H. 50, 75, and 100%. The effect of temperature on the ion conductivity of the ionomers is represented in Figure 1a. It shows that #1 (LSC + SSC, EW 1000) ionomer exhibits higher ion conductivity than #4 (LSC, EW 1000) ionomer, and #2 (LSC + SSC, EW 980) and #3 (SSC + SSC, EW 830) also demonstrate higher ion conductivity than #5 (SSC, EW 980) and #6 (SSC, EW 830) at all temperatures. It is in good agreement with the previous result that the presence of enhanced ion cluster channels in the blended ionomers led to an increase in the ion conductivity [39]. Figure 1b shows that #3 (SSC + SSC, EW 830) exhibits higher ion conductivity than the other five ionomers at 80 °C with R.H. 50, 75, and 100% due to the blending effect of ionomers and lower EW.

**Figure 2:**
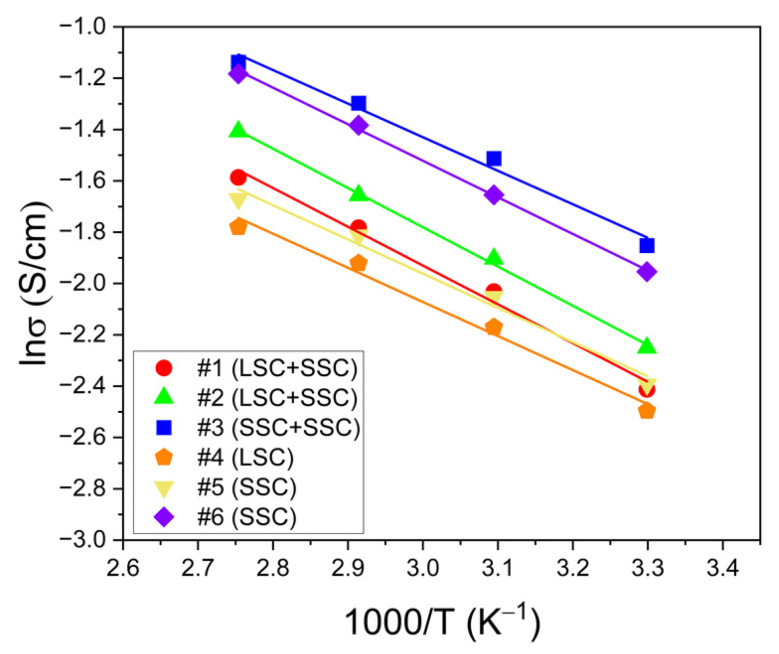
The temperature-dependent Arrhenius plots for the blended #1 (circle, LSC + SSC, EW 1000), #2 (triangle, LSC + SSC, EW 980), and #3 (square, SSC + SSC, EW 830) ionomers and the single #4 (pentagon, LSC, EW 1000), #5 (inverted triangle, SSC, EW 980), and #6 (diamond, SSC, EW 830) ionomers.

The activation energy of the ionomers representing the minimum energy required for proton conduction between ion cluster sites is shown in Figure 2. Low activation energy for proton conduction reduces energy loss due to the low ionic resistance of the ionomers and improves the PEMFC performance [40]. The activation energy of the ionomers is calculated based on the temperature-dependent ion conductivity using Arrhenius plots and is summarized in Table 2. The blended #3 (SSC + SSC, EW 830) shows the lowest activation energy value (10.86 kJ/mol), and the single #5 (SSC, EW 980) and #6 (SSC, EW 830) show values lower than 12 kJ/mol. The samples with low activation energy values were prepared using only the SSC-ionomer. The ionomers with SSC and low EW can help reduce the activation energy in proton conduction due to well-connected ion clusters and higher IEC compared to LSC-ionomers, respectively. Therefore, SSC-ionomers should be used for energy efficiency enhancements required for CL ionomer binders and membranes in PEMFCs [41].

**Figure 3:**
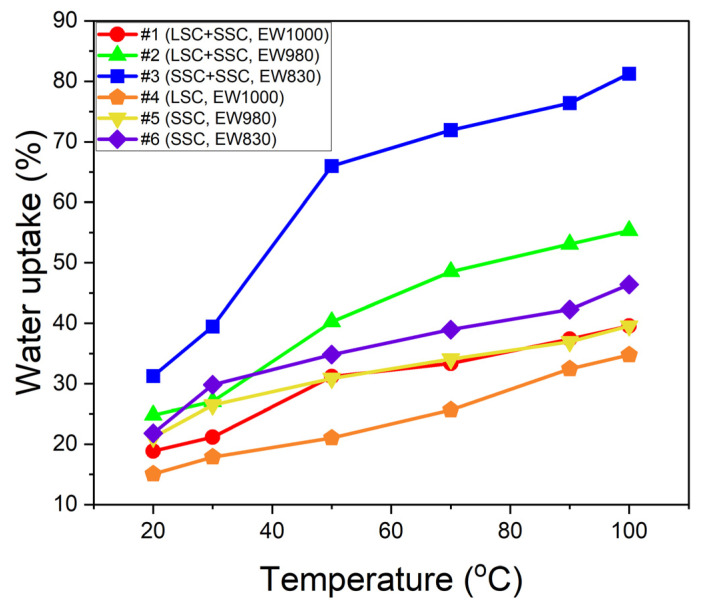
The water uptake of the blended #1 (circle, LSC + SSC, EW 1000), #2 (triangle, LSC + SSC, EW 980), and #3 (square, SSC + SSC, EW 830) ionomers and the single #4 (pentagon, LSC, EW 1000), #5 (inverted triangle, SSC, EW 980), and #6 (diamond, SSC, EW 830) ionomers.

Water uptake is an important ionomer’s property mainly influenced by the EW of the ionomers. Low-EW ionomers have a higher number of sulfonic acid groups, which results in a higher water uptake. Finally, it causes the formation of better ion clustering to enhance proton conduction. On the other hand, high-EW ionomers show the opposite behavior, which is lower water uptake and poor ion clustering [42]. The water uptake of the blended and single ionomers as a function of temperature is shown in Figure 3. The water uptake of all prepared ionomers increases with temperature, and the blended #1, #2, and #3 ionomers show higher water uptake than the single #4, #5, and #6 ionomers. It is noted that the blended #2 (LSC + SSC, EW 980) ionomer shows higher water uptake over the entire temperature range than the single #5 (SSC, EW 980) ionomer. In particular, the blended #3 (SSC + SSC, EW 830) ionomer shows a rapid increase in water uptake as the temperature increases. Compared to the single #6 (SSC, EW 830), much higher water uptake is exhibited. Even though they have a similar EW, it implies that the blended ionomers have a bulk structure to form a competent free volume. The movement of protons within ionomers depends on either the vehicular mechanism, which involves the transfer of hydrated protons and/or the Grotthuss mechanism, which entails the rearrangement and “hopping” of protons within extensive hydration structures [16,43]. The presence of well-formed ion cluster channels through the blended ionomers is attributed to improved water retention in the free volume of the ionomers. It is noteworthy for the PEMFC application as a membrane and/or CL binder in low external R.H. conditions due to their excellent water absorption.

The ORR requires reagents to approach through micro-pores in CLs and adsorb onto the ionomer–electrocatalyst surface, followed by a charge transfer between reactants and the ionomer–electrocatalyst surface and the resulting product to desorb from the surface. In this process, the cause of O_2_ diffusion limitations is a major issue for ORRs to be related to the oxygen flux through the thin film ionomer coating on the Pt catalyst particles or the micro-pores formed in CLs [9]. The oxygen permeability through the CL varies with the type of ionomers; in particular, EW and the type of side chain LSC ionomers strongly inhibit an ORR more than SSC ionomers due to dense coating on the electrocatalyst [24,25,44]. In addition, Yannick G. et al. reported that the SSC ionomer-based CL had a higher porosity than the LSC-based ionomer-based CL, primarily due to more uniform ionomer distributions in SSC-ionomer [45]. As a result, it exhibited less oxygen resistance and lower cathode proton transport resistance. The higher porosity of the SSC ionomer-based CL leads to an improvement in the performance and the current density within the mass transport region being limited [24,45,46,47].

**Figure 4:**
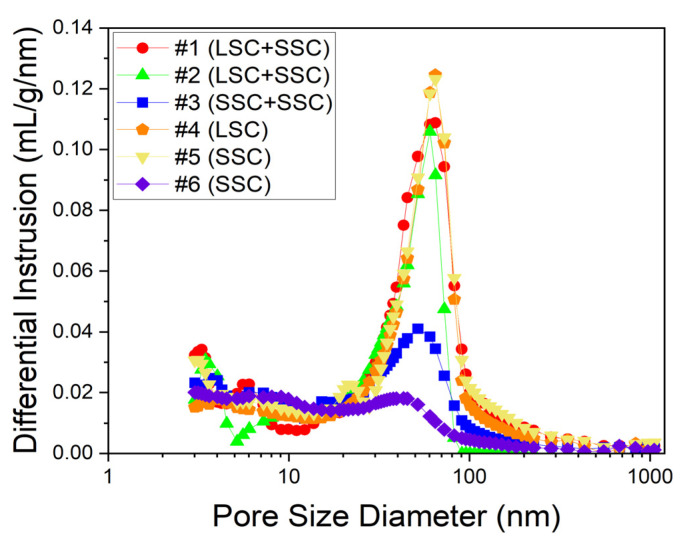
The pore diameter distribution of CLs prepared using the blended #1 (circle, LSC + SSC, EW 1000), #2 (triangle, LSC + SSC, EW 980), and #3 (square, SSC + SSC, EW 830) ionomers and the single #4 (pentagon, LSC, EW 1000), #5 (inverted triangle, SSC, EW 980), and #6 (diamond, SSC, EW 830) ionomers.

The porosity of CLs prepared by the blended and single ionomers was measured since the film morphology on the surface of electrocatalysts formed by the ionomer aggregates could determine the microstructure of CLs, mainly to influence O_2_ permeability. As shown in Figure 4, the measured porosity of the blended #1 (LSC + SSC, EW 1000), #2 (LSC + SSC, EW 980), and #3 (SSC + SSC, EW 830) ionomer-based CLs is 43.40, 46.85, and 45.38%, respectively, and that of the single #4 (LSC, EW 1000), #5 (SSC, EW 980), and #6 (SSC, EW 830) is 40.55, 42.18, and 35.73, respectively. It is observed that the blended ionomer-based CLs form higher porosity. Hence, it could be expected that the blended ionomer-based CLs with higher porosity help reduce the resistance of oxygen transport and attributes to lower mass transfer loss [48]. It is also noted that all the blended ionomers include SSC ionomers. As mentioned before, this is in good agreement with the previous results that the SSC ionomer-based CL had a higher porosity than the LSC-based ionomer-based CL [24,45,46,47].

The characterization and PEMFC performance of the MEAs was finally evaluated to prove the effect of the blended ionomer-based CLs with different water uptake and porosity on the microstructure of CLs. It could be influenced by the temperature of PEMFC operation and the R.H. particularly because the hydration and dehydration processes are involved in proton conduction and oxygen permeability within the cathodic CL. Insufficient proton conductivity and/or less oxygen permeability at an elevated temperature and/or low R.H. diminishes the CL activity [49,50]. In other words, the ionomer is a very important factor affecting the temperature and humidification conditions in relation to the performance and activity of the CL. Thus, it is important to design an ionomer capable of sufficient water management.

**Figure 5:**
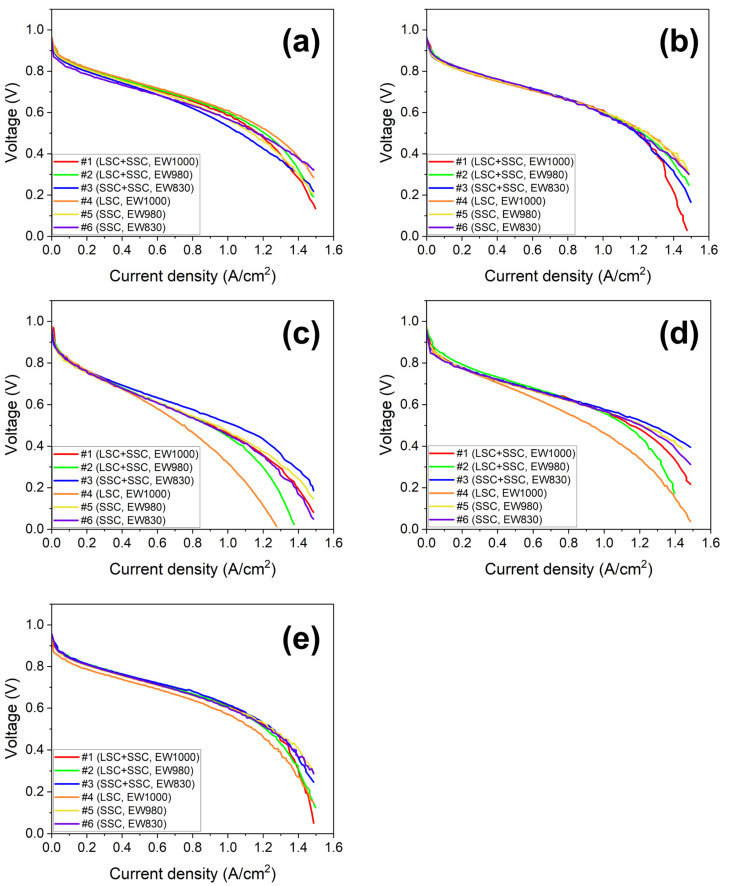
The I–V polarization of the MEAs based on the blended #1 (red, LSC + SSC, EW 1000), #2 (green, LSC + SSC, EW 980), and #3 (blue, SSC + SSC, EW 830) and the single #4 (orange, LSC, EW 1000), #5 (yellow, SSC, EW 980), and #6 (purple, SSC, EW 830) at the operation conditions: (**a**) 70 °C with R.H. 100%, (**b**) 75 °C with R.H. 100%, (**c**) 80 °C with R.H. 50%, (**d**) 80 °C with R.H. 75%, (**e**) 80 °C with R.H. 100% in a potentiostatic mode.

**Figure 6:**
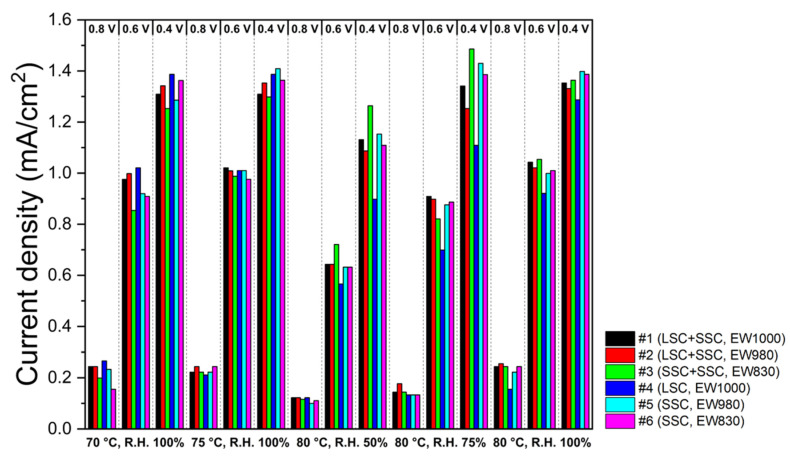
Current density from I-V polarization curves of the blended and the single ionomer-based MEAs measured at 70 °C with R.H. 100%, 75 °C with R.H. 100%, 80 °C with R.H. 50%, 80 °C with R.H. 75%, and 80 °C with R.H. 100%.

To evaluate fuel cell performance, the MEAs were fabricated using the blended ionomers and the single ionomers. The performance of the MEAs was evaluated at 70, 75, and 80 °C at R.H. 100% and R.H. 50 and 75% at 80 °C and was compared using the I–V polarization curves. The results of MEA evaluation are represented in Figure 5 and Figure 6. The PEMFC performance of both types (blended and single) of MEAs at 70 and 75 °C with R.H. 100% shows no difference at 0.4, 0.6, and 0.8 V. However, the superior performance of the blended ionomer-based MEAs is shown to that of the single ones at the elevated temperature (80 °C) and lower R.H. (50 and 75%). As discussed earlier, higher water uptake and porosity attribute to better performance. However, this behavior is not observed in comparison to ionomers with different EWs. As shown in Figure 1, two blended and single ionomers with EW 830 show the highest ion conductivity, which generally attains a higher PEMFC performance due to less Ohmic resistance. Nevertheless, the structure and some properties of nanoscale ionomer thin film are significantly different from the bulk, and its exact structure and behavior are still not precisely understood [9].

**Figure 7:**
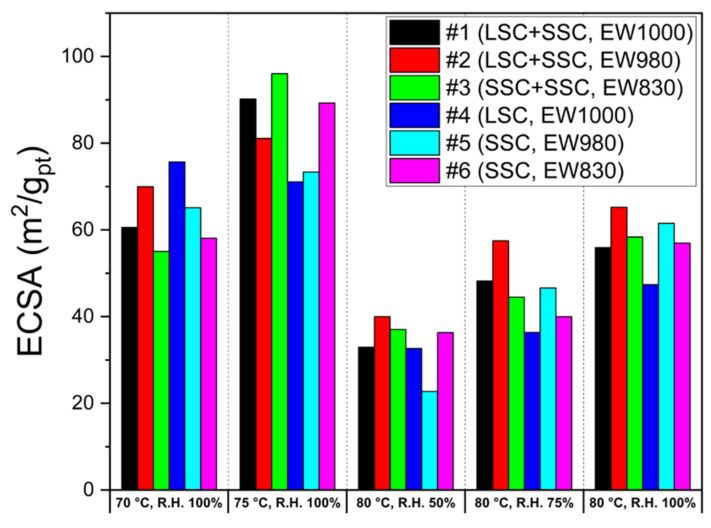
The ECSAs of the blended and the single ionomer-based MEAs from CVs measured at 70 °C with R.H. 100%, 75 °C with R.H. 100%, 80 °C with R.H. 50%, 80 °C with R.H. 75%, and 80 °C with R.H. 100%.

The ECSA results calculated from CVs measured under the same temperature and R.H. to I-V measurements are shown in Figure 7. The ECSA results show similar behavior to PEMFC performance. It is also shown that the ECSA of the blended ionomer-based MEAs is higher than that of the single ones at an elevated temperature (80 °C) and lower R.H. (50 and 75%). No distinguishable increase in ECSA at a lower EW has been revealed.

**Figure 8:**
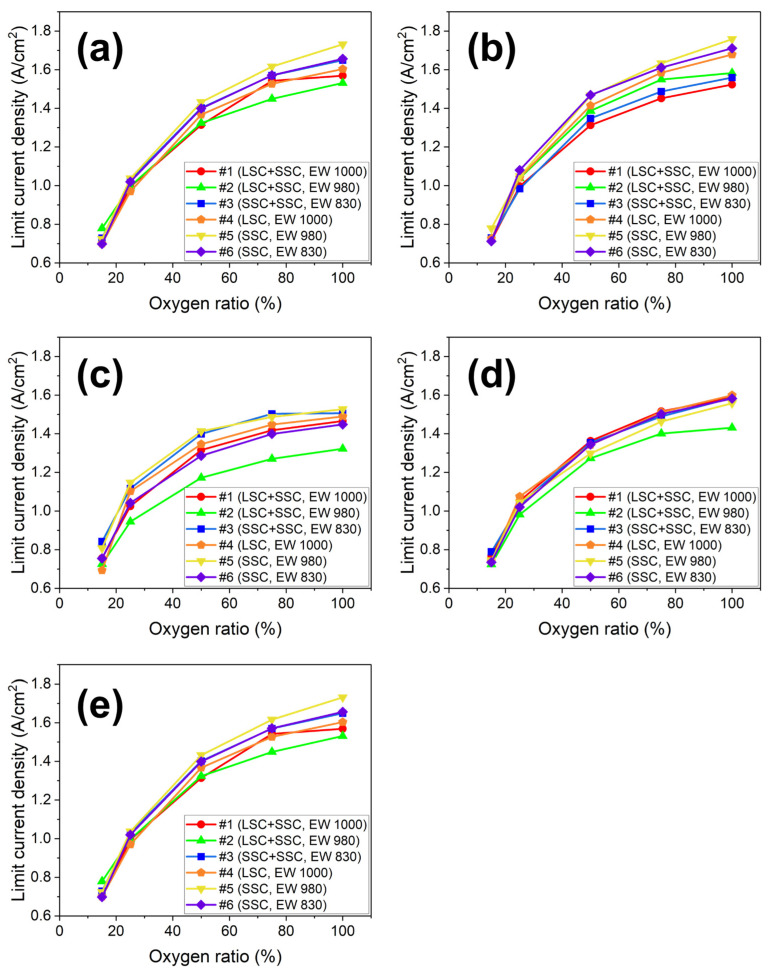
The oxygen ratio-dependent limiting current density of the blended and the single ionomer-based MEAs at (**a**) 70 °C with R.H. 100%, (**b**) 75 °C with R.H. 100%, (**c**) 80 °C with R.H. 50%, (**d**) 80 °C with R.H. 75%, (**e**) 80 °C with R.H. 100% for the blended #1 (circle, LSC + SSC, EW 1000), #2 (triangle, LSC + SSC, EW 980), and #3 (square, SSC + SSC, EW 830) ionomer and the single #4 (pentagon, LSC, EW 1000), #5 (inverted triangle, SSC, EW 980), and #6 (diamond, SSC, EW 830) ionomer.

**Figure 9:**
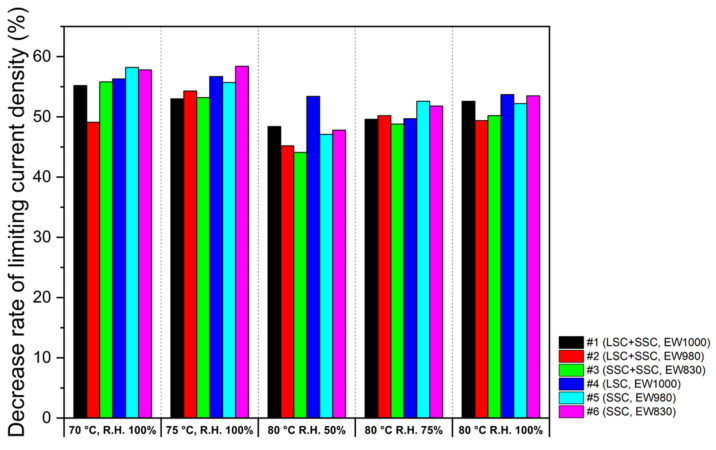
The decreasing rates of limiting current density of the blended and the single ionomer-based MEAs at 70 °C with R.H. 100%, 75 °C with R.H. 100%, 80 °C with R.H. 50%, 80 °C with R.H. 75%, and 80 °C with R.H. 100%.

The limiting current density in I–V polarization can be used to evaluate oxygen permeation resistance to CLs. It is reported that the smaller the reduction rate of the limiting current density with oxygen concentration, the lower the oxygen permeation resistance. [51,52,53]. The limiting current density reduction rate is defined by the difference in limiting the current density measured at maximum and minimum oxygen ratios. Figure 8 shows the decreasing rates of limiting current density obtained with oxygen concentration. In general, CLs with a high decreasing ratio have the disadvantage of gas permeation. The decreasing rates of limiting current density for two different types (blended and single) of MEAs are compared at 70, 75, and 80 °C at R.H. 100% and R.H. 50 and 75% at 80 °C. As shown in Figure 9, the results indicate that the blending ionomers exhibit lower decreasing rates in all the operation conditions due to the higher porosity of the blended ionomer-based CLs compared to the single ionomer-based CLs (Figure 4).

The blended ionomers have a higher water uptake and form a highly porous CL structure. Those characteristics result in an improved PEMFC performance and better mass transport, confirmed by the variation in I–V polarization, ECSA, and limiting current density. Compared to the single ionomer-based CLs, the blended ones could provide advantageous candidates for various PEMFC applications.

## 4. Conclusions

The optimization of CLs could be achieved by using sophisticated and integrated research efforts, such as the control of the CL morphology by ink composition, concentration, solvent, functionalization of electrocatalyst, and fabrication to attain higher Pt utilization and oxygen transport. This study is targeted to achieve a higher performance of CLs as a main component of fuel cell stack, which is still struggling to uncover scientific and commercial interests. The blended ionomers with EW 1000, 980, and 830 were prepared by the commercially available Nafion D1021, Nafion D2020, Aquivion D98-25BS, Aquivion D83-24BS, and Aquivion D72-25BS ionomer dispersions using a simple mixing method. Three different CLs based on the blended ionomers were investigated and correspondingly compared to single ionomer-based CLs with similar EW values. The blended ionomers positively influenced two aspects of the CLs microstructure. Firstly, the polymer properties of the blended ionomers were enhanced in terms of ion conductivity and water uptake. Secondly, the porosity of the CLs using blended ionomers increased to alleviate oxygen transport resistance. In the PEMFC evaluation, it was, hence, revealed that those effects significantly enhanced the fuel cell performance at the elevated temperature (80 °C) and lower R.H. (50 and 75%). Blended ionomer-based CLs with a higher water uptake and a porous CL structure result in an improved fuel cell performance and better mass transport than single ionomer-based CLs. In a future study, it is necessary to investigate the effect of the blended ionomers in CLs on durability. A study of their durability should be also performed with temperature and external humidification.

## Figures and Tables

**Table 1:** Experimental EW of the blended and single ionomers.

Theoretical EW of Ionomers (g/mol)	Experimental EW (g/mol)	
	Blended Ionomer	Single Ionomer
1000	1027 (#1, LSC + SSC)	1015 (#4, LSC)
980	937 (#2, LSC + SSC)	998 (#5, SSC)
830	832 (#3, SSC + SSC)	866 (#6, SSC)

**Table 2:** The activation energy of the blended and single ionomers calculated using Arrhenius plots.

Theoretical EW of Ionomers (g/mol)	Activation Energy (kJ/mol)	
	Blended Ionomer	Single Ionomer
1000	12.55 ± 0.0117 (#1, LSC + SSC)	11.04 ± 0.202 (#4, LSC)
980	12.72 ± 0.153 (#2, LSC + SSC)	11.09 ± 0.211 (#5, SSC)
830	10.86 ± 0.167 (#3, SSC + SSC)	11.84 ± 0.105 (#6, SSC)

## Data Availability

Data sharing not applicable.
